# Depression Screening for the Geriatric Population Visiting Primary Healthcare Centers in the Eastern Region of Saudi Arabia

**DOI:** 10.7759/cureus.17971

**Published:** 2021-09-14

**Authors:** Abdulrhman Alabdulgader, Ali O Mobarki, Ahmed AlDuwayrij, Abdullah Albadran, Mohammed I Almulhim, Abdullah Almulhim

**Affiliations:** 1 Internal Medicine, King Abdulaziz Medical City Riyadh, Ministry of the National Guard-Health Affairs, Riyadh, SAU; 2 Medicine and Surgery, King Faisal University, Al Ahsa, SAU; 3 College of Medicine, King Faisal University, Al Ahsa, SAU; 4 Family Medicine, King Faisal University, Al Ahsa, SAU

**Keywords:** screening, primary health care, phq-9, elderly, prevalence, depression

## Abstract

Background

Depression is considered one of the most common psychiatric disorders that affects more than 260 million people in all age groups worldwide. Yet, among the geriatric population, in which it can show nonspecific symptoms, depression can be easily underdiagnosed. The objectives of this study are to assess the prevalence of depression among the geriatric population in primary healthcare centers and to estimate the effects of different sociodemographic and medical factors.

Methodology

A total of 408 patients aged 60 years or older were approached in the primary healthcare centers of the eastern region of Saudi Arabia. Using the Patient Health Questionnaire-9, patients were either interviewed or filled the questionnaire by themselves. Questions about sociodemographic data and medical and medication histories were included in the questionnaire.

Results

Of the 408 participants, 173 (42.4%) reported depressive symptoms; 115 (28.2%) of the participants had mild depression, 50 (12.3%) had moderate depression, and 8 (2%) reported moderately severe depression. Correlates of depression included elderly patients aged 75 years or more, of whom 78.9% showed depression compared to 39.3% of those who were 60-65 years old (P = 0.001). Furthermore, the female elderly showed higher rates of depression compared to males (52.8% vs. 35.7%; P = 0.001). A reported 81.1% of the elderly were diagnosed with chronic diseases; approximately half of them were depressed, while only 32.9% of the elderly free of diseases were depressed (P = 0.001).

Conclusions

The prevalence of depression is high among the elderly in the eastern province of Saudi Arabia, especially in those who complain of chronic diseases, older patients, and females. Screening for depression must be employed early to manage depressive symptoms and prevent further complications.

## Introduction

Depression is the most common mood disorder which affects all age groups [1]. More than 260 million individuals around the world suffer from depression [2]. The World Health Organization (WHO) has reported that depression is the fourth most common disabling disease in the world [3]. Furthermore, depression is considered a strong risk factor for potential suicides [4]. Elements that appear to be associated with depression are lack of physical activities, medical history, social connections, illicit drug use, and alcohol intake [5]. Depressed patients can present with low mood, poor sleep, loss of pleasure, fatigue, impaired concentration, and feelings of guilt [6].

Several previous studies have shown that depression symptoms are more encountered in the geriatric population [7-9]. Unfortunately, a large number of depression cases in older people are easily missed because many of them present with nonspecific symptoms and few get the chance to be treated [10]. In the medical literature, several screening instruments have been developed to screen depression. Of these, the easiest and most reliable screening tool is the nine-item Patient Health Questionnaire (PHQ-9) [11], which was proven in several meta-analyses to have high sensitivity and specificity in primary care settings and is considered by some to be the gold-standard screening instrument for primary care patients who present with depressive symptoms [12]. In Saudi Arabia, depression screening has been assessed in several studies, with detection rates varying from 10% to 20% [7,13]. Unfortunately, the prevalence of geriatric depression in the eastern region of Saudi Arabia has not been documented. Our main aim, therefore, is to screen the geriatric population visiting primary health centers for depression to determine its estimated prevalence and possible associated factors in the eastern region of the Kingdom of Saudi Arabia.

Research objectives

To estimate the prevalence of geriatric depression in primary healthcare centers in the eastern region of Saudi Arabia. To evaluate the relationship of depression to sociodemographic variables and medical and medication histories.

## Materials and methods

Study design and participants

This study employed a cross-sectional design. Our study participants were geriatric patients who visited primary health centers during the five months from February 2021 to June 2021 in the largest region of Saudi Arabia, the eastern region. We included all elderly patients who fulfilled the following inclusion criteria: (i) aged 60 or above, (ii) able to understand and speak Arabic or English, and (iii) intellectually capable. Patients with impaired cognition, acute psychosis, schizophrenia, bipolar disorder, unipolar depression, language barrier, aphasia, hearing impairment, reduced level of consciousness, or unstable medical illnesses were excluded from the study. All eligible patients were invited to participate in the study. Ethical approval was obtained from the institutional review board of the College of Medicine, King Faisal University (2020-12-27). An Arabic or English informed consent form was signed by the participants before the interviews. Ethical considerations and confidentiality of the participants’ information were maintained throughout the study by making the participants’ information anonymous.

Sample size and technique

We calculated the required sample size to be 385 convenient participants from primary healthcare centers in the eastern province of Saudi Arabia, with a confidence interval of 95% and a margin of error of 5% [14].

Data collection method

After they provided their informed consent, participants who met our criteria completed the questionnaire, which consisted of two parts: (i) sociodemographic data and medical and medication histories, and (ii) the PHQ-9, which is one of the most commonly used depression screening instruments in primary health centers. The PHQ-9 was reported to be the most sensitive depression screening instrument in a meta-analysis of 113 studies, demonstrating 84% sensitivity and 88% specificity [15]. Previous studies have confirmed that it is valid and reliable in Arabic [11,16]. The PHQ-9 questionnaire consists of nine questions [11] that ask participants about the following: loss of interest, feelings of hopelessness, sleeping abnormalities, feeling tired, appetite abnormalities, feeling bad about oneself, trouble concentrating on things, moving or speaking very slowly or extreme restlessness, and thoughts that you would be better off dead or of hurting oneself in some way. Each item is scored on a scale of 0 (not at all) to 3 (nearly every day), with the total score ranging from 0 to 27. A score of 5 to less than 10 indicates mild severity, 10 to less than 15 indicates moderate severity, 15 to less than 20 indicates moderately severe disease, and 20 to 27 indicates severe disease.

Statistical analysis

The data were collected, reviewed, and analyzed using the Statistical Package for Social Sciences version 21 (IBM Corp., Armonk, NY). All statistical methods used were two-tailed with an alpha level of 0.05 and were considered significant if the P-value was less than or equal to 0.05. As for the PHQ-9, the overall scores, the cutoff ranged from 0-19 points, were obtained by summing all discrete scores for the items. Elderly classification according to depression severity was applied in reference to the cutoff points, while a descriptive analysis was done by describing the frequency distribution and percentage of the study variables, including patients’ biodemographic data, PHQ-9 items, and depression prevalence and severity. A cross-tabulation for showing the distribution of elderly depression according to their biodemographic data was carried out using Pearson’s chi-square test for significance. To assess the most significant predictors for having depression among the elderly participants, multiple logistic models were applied to assess the adjusted likelihood for developing depression.

## Results

A total of 503 questionnaires were distributed; 35 (6.9%) refused to participate, 54 (10.7%) were excluded based on our exclusion criteria, and 6 (1.1%) were rejected due to incomplete questionnaires. The study included 408 eligible elderly from 25 primary healthcare centers, whose ages ranged from 60 to 87 years with a mean age of 63.5 ± 11.9 years. Of them, 249 (61%) were males. Chronic illness was reported by 331 (81.1%) of the participants; the most reported illness was diabetes mellitus 221 (54.2%), followed by hypertension 196 (48%), obesity 70 (17.2%), cardiovascular diseases 34 (8.3%), and hepatic diseases 8 (2%). Concerning the participants’ medications, 204 (50%) were on antidiabetic medication, 188 (46.1%) were taking antihypertensive drugs, and 110 (27%) were on insulin. Overall, 83 (20.3%) participants were not taking any drugs (Table 1).

**Table 1:** Biodemographic data of the geriatric population visiting primary healthcare centers in the eastern region of Saudi Arabia. DM: diabetes mellitus; HTN: hypertension; CVD: cardiovascular disease

Biodemographic data	No	%
Age in years		
60–65	244	59.8%
66–70	94	23.0%
71–75	51	12.5%
>75	19	4.7%
Gender		
Male	249	61.0%
Female	159	39.0%
Chronic diseases		
None	73	17.9%
DM	221	54.2%
HTN	196	48.0%
Obesity	70	17.2%
Atherosclerosis	20	4.9%
CVD	34	8.3%
Stroke	10	2.5%
Chronic respiratory diseases	26	6.4%
Renal diseases	22	5.4%
Hepatic diseases	8	2.0%
Others	16	3.9%
Received drugs		
None	83	20.3%
Antidiabetic	204	50.0%
Antihypertensive	188	46.1%
Insulin	110	27.0%
Bronchodilators	27	6.6%
Cortisone	6	1.5%
Antidepressant	1	0.2%
Others	14	3.4%

Table 2 shows the frequency of PHQ-9 items among the study participants. A total of 243 (59.6%) participants reported that they felt tired or had little energy to some degree; 231 (56.6%) reported that they had trouble falling or staying asleep, or slept too much; 186 (45.6%) had either poor appetite or would overeat; and 175 (42.9%) felt down, depressed, or hopeless. Furthermore, 134 (32.8%) had trouble concentrating on things, such as reading the newspaper or watching television, while only 56 (13.7%) reported moving or speaking so slowly that other people could notice or being so fidgety or restless that they moved around a lot more than usual. Only 21 (5.1%) participants had thoughts that they would be better off dead or of hurting themselves in some way. These feelings were somewhat difficult among 28 (6.9%) and extremely difficult among 9 (2.2%) participants, but not difficult at all among 254 (62.3%) participants.

**Table 2:** Frequency of PHQ-9 items among study participants, eastern region, Saudi Arabia. PHQ-9: Patient Health Questionnaire-9

PHQ-9 items		No	%
Little interest or pleasure in doing things	Not at all	294	72.1%
	Several days	79	19.4%
	More than half the days	19	4.7%
	Nearly every day	16	3.9%
Feeling down, depressed, or hopeless	Not at all	233	57.1%
	Several days	133	32.6%
	More than half the days	27	6.6%
	Nearly every day	15	3.7%
Trouble falling or staying asleep, or sleeping too much	Not at all	177	43.4%
	Several days	119	29.2%
	More than half the days	53	13.0%
	Nearly every day	59	14.5%
Feeling tired or having little energy	Not at all	165	40.4%
	Several days	148	36.3%
	More than half the days	50	12.3%
	Nearly every day	45	11.0%
Poor appetite or overeating	Not at all	222	54.4%
	Several days	122	29.9%
	More than half the days	38	9.3%
	Nearly every day	26	6.4%
Feeling bad about yourself—or that you are a failure or have let yourself or your family down	Not at all	315	77.2%
	Several days	79	19.4%
	More than half the days	7	1.7%
	Nearly every day	7	1.7%
Trouble concentrating on things such as reading the newspaper or watching television	Not at all	274	67.2%
	Several days	100	24.5%
	More than half the days	20	4.9%
	Nearly every day	14	3.4%
Moving or speaking so slowly that other people could have noticed? Or the opposite—being so fidgety or restless that you have been moving around a lot more than usual	Not at all	352	86.3%
	Several days	40	9.8%
	More than half the days	9	2.2%
	Nearly every day	7	1.7%
Thoughts that you would be better off dead or of hurting yourself in some way	Not at all	387	94.9%
	Several days	20	4.9%
	More than half the days	0	0.0%
	Nearly every day	1	0.2%
If you checked off any problems, how difficult have these problems made it for you to do your work, take care of things at home, or get along with other people?	Not difficult at all	254	62.3%
	Somewhat difficult	117	28.7%
	Very difficult	28	6.9%
	Extremely difficult	9	2.2%

**Figure 1:**
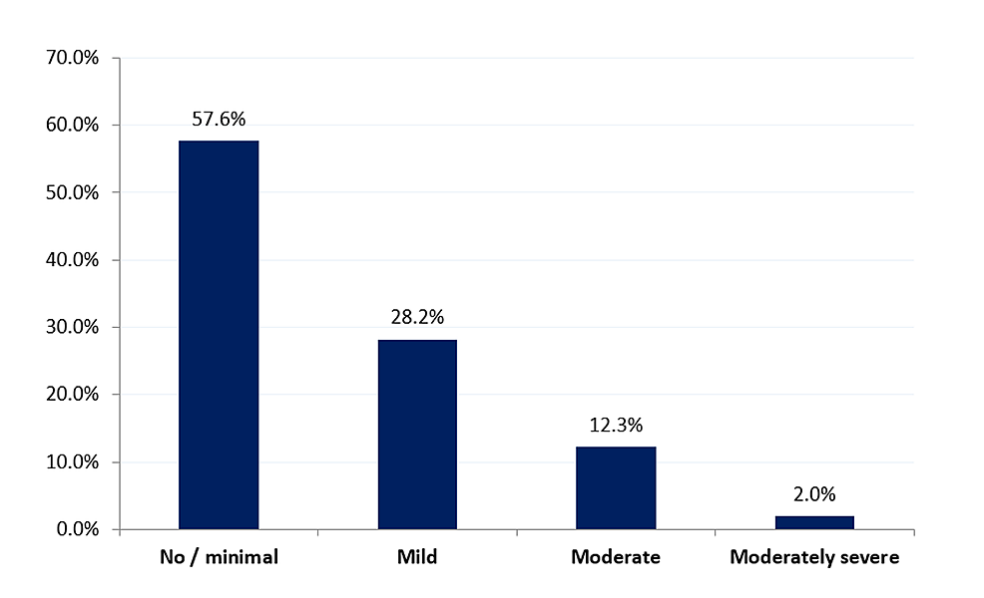
Prevalence and severity of depression among the geriatric population visiting primary healthcare centers in the eastern region of Saudi Arabia.

Figure 1 illustrates the prevalence and severity of depression among the geriatric population visiting primary healthcare centers in the eastern region of Saudi Arabia. A total of 173 (42.4%) participants reported suffering from depression, which was mild for 115 (28.2%), moderate for 50 (12.3%), and moderately severe for 8 (2%) respondents.

Table 3 reveals the distribution of depression among the study participants according to their biodemographic data. Overall, 78.9% of elderly respondents aged 75 or more showed depression compared to 39.3% aged 60-65 years with statistical significance (P = 0.001). In addition, depression was significantly higher among female compared to male respondents (52.8% vs. 35.7%; P = 0.001). Among those who had hepatic diseases, 6 (75%) had depression compared to 14 (70%) who complained of atherosclerosis and 21 (61.8%) who had cardiovascular diseases, while only 24 (32.9%) who were free of disease were depressed (P = 0.001). Medications were insignificantly associated with the depression rate among the elderly respondents (P = 0.062).

**Table 3:** Distribution of depression among the elderly according to their biodemographic data, eastern region, Saudi Arabia. P: Pearson’s chi-square test; * P < 0.05 (significant) DM: diabetes mellitus; HTN: hypertension; CVD: cardiovascular disease

Biodemographic data	Depression				P-value
	No		Yes		
	No	%	No	%	
Age in years					0.001*
60–65	148	60.7%	96	39.3%	
66–70	64	68.1%	30	31.9%	
71–75	19	37.3%	32	62.7%	
>75	4	21.1%	15	78.9%	
Gender					0.001*
Male	160	64.3%	89	35.7%	
Female	75	47.2%	84	52.8%	
Chronic diseases					0.001*
None	49	67.1%	24	32.9%	
DM	125	56.6%	96	43.4%	
HTN	103	52.6%	93	47.4%	
Obesity	28	40.0%	42	60.0%	
Atherosclerosis	6	30.0%	14	70.0%	
CVD	13	38.2%	21	61.8%	
Stroke	4	40.0%	6	60.0%	
Chronic respiratory diseases	11	42.3%	15	57.7%	
Renal diseases	11	50.0%	11	50.0%	
Hepatic diseases	2	25.0%	6	75.0%	
Others	5	31.3%	11	68.8%	
Received drugs					0.062
None	54	65.1%	29	34.9%	
Antidiabetic	115	56.4%	89	43.6%	
Antihypertensive	101	53.7%	87	46.3%	
Insulin	65	59.1%	45	40.9%	
Bronchodilators	11	40.7%	16	59.3%	
Cortisone	1	16.7%	5	83.3%	
Antidepressant	0	0.0%	1	100.0%	
Others	6	42.9%	8	57.1%	

Table 4 shows a multiple logistic regression model for predictors of depression among the study participants. Among the included factors, age and gender were the most adjusted significant predictors of depression. The elderly aged 71-75 had nearly triple the risk (odds ratio [OR] = 2.7) for developing depression than younger age groups, while the elderly aged over 75 (OR = 5.38) showed five times the risk. Female respondents had double the risk for developing depression than male (OR = 2.2) respondents, keeping all other factors constant.

**Table 4:** Multiple logistic regression model for predictors of depression among the study participants, eastern region, Saudi Arabia. OR_a_: adjusted odds ratio; CI: confidence interval; * P < 0.05 (significant)

Factors	OR_a_	95% CI		P-value
		Lower	Upper	
Age group in years				0.000
66–70	0.67	0.40	1.13	0.136
71–75	2.71	1.41	5.20	0.003*
>75	5.38	1.70	17.01	0.004*
Female gender	2.22	1.45	3.39	0.000*
Had chronic disease	1.98	0.48	8.13	0.342
Receiving drugs	0.77	0.20	2.92	0.700
Pseudo R^2^; model significance	0.21; 0.001*			
Model accuracy	71%			

## Discussion

This study provides the first data-based estimate of the prevalence of depression in the geriatric population attending primary healthcare centers in Saudi Arabia in the twenty-first century. In our findings, the overall prevalence of undiagnosed depressive symptoms in elderly participants attending primary healthcare centers was 42.4% based on the PHQ-9 criteria. These findings are higher compared to previous studies conducted in Saudi Arabia, China, Brazil, and Germany, which found an overall prevalence of 39%, 30.65%, 26.1%, and 9.7%, respectively [8,17-19]. Previous studies have determined that major depressive disorder is a common finding in geriatric populations, with a prevalence reaching up to 10% of their sample [20]. Fortunately, only 2% of our study participants were classified as having moderately severe depression, while two-thirds of depressed respondents (28.2%) who were mildly depressed were the most common subtype in our results. This prevalence of depressive symptoms may be overestimated compared with other studies because we included any elderly individual who met our inclusion criteria attending the primary healthcare centers, which resulted in a high detection rate of depressive symptoms, unlike the study conducted in Germany, which excluded patients who had multiple comorbidities living in long-term care facilities [19].

The low prevalence of moderately severe depression in our study may be attributed to the religious, cultural aspects, and strong social bonds between the elderly and their family members [21]. Of the 81.1% of our sample who complained of chronic diseases, about half were depressed. On the other hand, only 32.9% of the elderly free of diseases were depressed. Our analysis showed that the prevalence of depression among participants with comorbid chronic diseases was significantly higher than participants who were free of chronic diseases, which is consistent with other studies [19]. The high number of comorbid diseases in our sample is mainly attributed to the lack of screening programs in primary healthcare centers in Saudi Arabia, including but not limited to diabetes mellitus, hypertension, atherosclerosis, etc. [22]. Participants aged 71-75 had nearly triple the risk for developing depression than those less old. However, in another study, the prevalence of depressive symptoms in the Saudi young population was 49.9%, which is higher than the symptoms in our elderly population [23]. These differences may be explained by the growing difficulties facing the young in this era, particularly in the form of career and financial stressors. In addition, one of the significant predictors for having depression in our study was being female. Women had double the risk of developing depression than men. Comparison of this finding with other studies confirms that depression is significantly more common in women than in men [7,17]. Surprisingly, depression was not significantly associated with antidiabetics, antihypertensives, bronchodilators, or any other medication. This finding is contrary to previous studies which have suggested that drugs are significantly associated with depression [7]. Further studies may either confirm or refute this variation. Interestingly, thoughts of being better off dead or self-harm were experienced by 5.1% of the participants. This is similar to another study conducted in the central region of Saudi Arabia among participants aged less than 65, which found that 3.7% of the young had experienced such thoughts [23].

Limitations

In this study, several factors may have influenced the findings and resulted in some limitations. First, our results are based on a cross-sectional analysis and therefore the causality between depressive symptoms and their correlates is not definitive. Second, anxiety and mild cognitive impairment patients were excluded based on self-report, which may be a confounding factor. Third, although the PHQ-9 is a brief and reliable screening tool for depression, further assessments are required by psychiatrists to determine a patient’s definitive diagnosis and develop a management plan.

## Conclusions

Our results indicate that depression is prevalent among the elderly in the eastern region of Saudi Arabia, in particular among the elderly with chronic illnesses such as diabetes mellitus, hypertension, and cardiovascular diseases. We found that depression is highly associated with age as the risk increases with older age. Furthermore, women had double the risk than men for developing depression. These study findings should encourage primary healthcare centers to investigate depression carefully among the elderly using screening instruments such as the PHQ-9. Screening for depression using such criteria is important to detect depression early, which will prevent further medico-socio-psychological complications.
